# Human *β*-Defensin 3 Reduces TNF-*α*-Induced Inflammation and Monocyte Adhesion in Human Umbilical Vein Endothelial Cells

**DOI:** 10.1155/2017/8529542

**Published:** 2017-02-28

**Authors:** Tianying Bian, Houxuan Li, Qian Zhou, Can Ni, Yangheng Zhang, Fuhua Yan

**Affiliations:** ^1^Department of Periodontology, Nanjing Stomatological Hospital, Medical School of Nanjing University, Nanjing, Jiangsu, China; ^2^Central Laboratory of Stomatology, Nanjing Stomatological Hospital, Medical School of Nanjing University, Nanjing, Jiangsu, China; ^3^Department of Endodontics, Nanjing Stomatological Hospital, Medical School of Nanjing University, Nanjing, Jiangsu, China

## Abstract

The aim of this study was to investigate the role of human *β*-defensin 3 (hBD3) in the initiation stage of atherosclerosis with human umbilical vein endothelial cells (HUVECs) triggered by tumor necrosis factor- (TNF-) *α*. The effects of hBD3 on TNF-*α*-induced endothelial injury and inflammatory response were evaluated. Our data revealed that first, hBD3 reduced the production of interleukin-6 (IL-6), IL-8, monocyte chemoattractant protein-1 (MCP-1), and macrophage migration inhibitory factor (MIF) in HUVECs in a dose-dependent manner. In addition, hBD3 significantly prevented intracellular reactive oxygen species (ROS) production by HUVECs. Second, western blot analysis demonstrated that hBD3 dose-dependently suppressed the protein levels of intracellular adhesion molecule-1 (ICAM-1) and vascular cell adhesion molecule-1 (VCAM-1) in TNF-*α*-induced HUVECs. As a result, hBD3 inhibited monocyte adhesion to TNF-*α*-treated endothelial cells. Additionally, hBD3 suppressed TNF-*α*-induced F-actin reorganization in HUVECs. Third, hBD3 markedly inhibited NF-*κ*B activation by decreasing the phosphorylation of IKK-*α*/*β*, I*κ*B, and p65 subunit within 30 min. Moreover, the phosphorylation of p38 and c-Jun N-terminal protein kinase (JNK) in the mitogen-activated protein kinase (MAPK) pathway were also inhibited by hBD3 in HUVECs. In conclusion, hBD3 exerts anti-inflammatory and antioxidative effects in endothelial cells in response to TNF-*α* by inhibiting NF-*κ*B and MAPK signaling.

## 1. Introduction

Atherosclerosis is a vascular inflammatory disease characterized by the accumulation of lipids and immune cells on the inner face of the arterial wall. Inflammatory stimulation, even low level endotoxemia, can aggravate the progression of this disease [1]. A monolayer of endothelial cells, which directly contacts the flowing blood, is easily attacked by various stimulating factors. Endothelial cell dysfunction triggered by such stimulations is believed to be the initiation step and key contributor in the pathogenesis of atherosclerosis [2]. Endothelial cells at sites of inflammation are not only participants but also regulators of inflammation [3, 4]. During inflammation, a large amount of inflammatory mediators are produced by immune cells, such as macrophages, and endothelial cells are the main targets. Then, the activated endothelial cells secrete a broad spectrum of cytokines and chemokines, recruiting monocytes. Enhanced migration and adhesion of monocytes to the endothelium are suggested to play a crucial role in atherosclerosis. In addition, the expression of adhesion molecules required for the firm binding of monocytes is also upregulated on endothelial cell surfaces [5].

In our previous study, the results demonstrated that chronic exposure to the endotoxin of* Porphyromonas gingivalis* can aggravate atherosclerotic lesions in ApoE-deficient mice [6]. Indeed, compelling evidence has demonstrated the proatherogenic role of inflammatory cytokines in the pathogenesis of atherosclerosis. Elevated levels of several inflammatory markers in the circulation are risk factors for atherosclerotic events [7]. Tumor necrosis factor-*α* (TNF-*α*), one of the most potent inflammatory cytokines, is closely associated with atherosclerosis. It is mainly produced by activated macrophages. It is a pleiotropic cytokine mediating inflammation, immunity, and apoptosis. For vascular endothelial cells, exposure to TNF-*α* triggers several signaling cascades in human umbilical vein endothelial cells (HUVECs), especially the nuclear factor *κ*B (NF-*κ*B), c-Jun N-terminal kinase (JNK), and p38 mitogen-activated protein kinase pathways, leading to the production of inflammatory cytokines [8]. In addition, TNF-*α* can reorganize the F-actin cytoskeleton of endothelial cells, leading to the formation of stress fibers [9]. TNF-*α* can also modulate EC permeability by enlarging intercellular gaps, promoting vascular leakage at sites of inflammation [10].

TNF-*α* stimulation is also associated with increased intracellular levels of reactive oxygen species (ROS) formation, enhancing monocyte recruitment and adhesion to the vascular endothelium. This process is mediated mainly by elevated cell adhesion molecules such as intercellular adhesion molecule-1 (ICAM-1), vascular cell adhesion molecule-1 (VCAM-1), and endothelial cell selectin (E-selectin).

Defensins are naturally occurring peptides with a wide range of antimicrobial, antiviral, and immunomodulatory properties. They are cationic, cysteine-rich, *β*-sheet, and tridisulfide peptides. In humans, defensins are classified into two categories, *α*- and *β*-defensins. *β*-defensins are mainly produced by the mucosa and epithelial cells. Intraocular tissues and the human endometrium are also reported to express *β*-defensins [11]. Several studies have documented the participation of antimicrobial peptides in endothelial immune defense [12, 13]. In addition, *β*-defensins are beneficial for wound healing of endothelial cells [14]. Human *β*-defensin 3 (hBD3) is a member of the *β*-defensin family. In addition to epithelial cells, hBD3 is also expressed in endothelial cells. The gene and protein expression of hBD3 are induced in human umbilical vein endothelial cells simulated with TNF-*α* [15]. The upregulation of hBD3 by TNF-*α* in HUVECs illustrates the important role that defensins play in host immune defense against inflammation. In our previous study, we demonstrated that hBD3 strongly inhibited the progression of early-stage atherosclerotic lesions and inflammation levels in RAW 264.7 cells and human THP-1-derived macrophages [16, 17].

However, the cell types targeted by hBD3 might not be restricted to macrophages. Vascular endothelial cells and monocytes/macrophages are both atherogenic and critical components in the process of atherogenesis [18]. The interaction between macrophages and endothelial cells is very crucial in atherosclerosis. In normal conditions, resting endothelial cells form an integrated barrier at the blood-tissue interface. Upon inflammatory stimulation, quiescent endothelial cells may transform into a proatherogenic phenotype, inducing the infiltration of monocytes [19]. The recruited monocytes then differentiate into macrophages, contributing to a vicious cycle promoting endothelial dysfunction [20]. As the endothelium has already been a therapeutic target, hBD3 might also manifest its protective effects by interacting with vessel endothelial cells. The effects of hBD3 on TNF-*α*-induced endothelial cell activation remain unknown.

Thus, the present study was designed to focus on the effects of hBD3 on TNF-*α*-stimulated endothelial cell dysfunction, including the enhanced production of inflammatory mediators, monocyte adhesion, and the expression of cell adhesion molecules, all of which are early events in the pathogenesis of atherosclerosis.

## 2. Materials and Methods

### 2.1. Cell Culture and Reagents

Human primary umbilical vein endothelial cells (HUVECs) (ScienCell Research Laboratories, San Diego, CA) were maintained in endothelial cell medium (ECM) (ScienCell Research Laboratories) supplemented with 5% FBS, 1% penicillin/streptomycin, and 1% endothelial cell growth supplement (ECGS). For the present study, cells at passages 3–6 were seeded and grown until confluence.

Recombinant human TNF-*α* and hBD3 were obtained from PeproTech (Rocky Hill, NJ, USA). Anti-ICAM-1 and anti-VCAM-1 antibodies were obtained from Santa Cruz Biotechnology (Santa Cruz, CA, USA). Antiphosphorylated and total I*κ*B*α*, p65, p38, ERK, and JNK antibodies were purchased from Cell Signaling Technology (Beverly, MA).

### 2.2. Cell Viability Assay

The cell viability of HUVECs was determined using a CCK8 assay according to the manufacturer's instructions. HUVECs (5000 cells/well) were prepared in 96-well plates and incubated overnight before treatment with various concentrations of hBD3 (20, 10, 5, 2.5, or 1.25 *μ*g/mL) and TNF-*α* (40 ng/mL) for 24 h. Subsequently, 10 *μ*L of CCK8 solution (Dojindo Laboratories, Kumamoto, Japan) was added to each well, and the cells were incubated for an additional 3 h. The optical density of each well was evaluated at a wavelength of 450 nm on a microplate spectrophotometer.

### 2.3. Enzyme-Linked Immunosorbent Assay (ELISA)

HUVECs were seeded and cultured in a 96-well plate until confluent. The control group was treated with ECM and the other seven groups were treated with TNF-*α* (40 ng/mL) in the presence of various concentrations of hBD3 for 24 h. Then the cell supernatants were collected by centrifugation. The assay was performed with specific ELISA kits (R&D Systems) according to the manufacturer's recommendations.

### 2.4. Monocyte Adhesion Assay

Confluent HUVECs were stimulated with TNF-*α* (40 ng/mL) in the presence or absence of hBD3 for 24 h. After that, the cells were washed with RPMI-1640 medium before being cocultured with THP-1 cells. THP-1 cells were prelabeled with 2 *μ*M calcein-AM (Dojindo Laboratories) in RPMI-1640 medium for 20 min in the cell incubator before being added to each well of HUVECs and further incubated for 30 min. After coincubation, each well was washed gently and thoroughly with RPMI-1640 medium supplemented with 1% FBS to remove the nonadherent THP-1 cells. The attached THP-1 cells were examined under a microscope (Olympus Corporation, Tokyo, Japan) and fluorescence microplate reader at excitation and emission wavelengths of 490 nm and 515 nm.

### 2.5. Western Blot Analysis

The cells were lysed in RIPA buffer supplemented with protease inhibitors for 30 min, and the whole cell lysates were separated by electrophoresis in sodium dodecyl sulfate polyacrylamide gels (SDS-PAGE) and then transferred to PVDF membranes (Millipore, Bedford, MA, USA). The membranes were blocked with 5% BSA at room temperature for 2 h before incubation overnight with primary antibodies against the target protein. Blots were washed with Tris-buffered saline containing 0.1% Tween 20 three times and then incubated for 2 h with anti-rabbit secondary antibody at room temperature. The bands were detected using chemiluminescence detection agents. Images were captured with an ImageQuant LAS 4000 digital imaging system (GE Healthcare, Piscataway, NJ).

### 2.6. Immunofluorescence Analysis

HUVECs were seeded onto sterile glass coverslips in a 12-well cell culture plate. After incubation overnight, the cells were treated with the indicated concentrations of hBD3 under TNF-*α* stimulation for the indicated time. Subsequently, the cells were washed and fixed in 4% paraformaldehyde for 20 min at room temperature and then treated with 0.3% Triton X-100 for 15 min. After that, the cells were blocked with 3% BSA for 1 h at room temperature. Then, the cells were further incubated with the indicated rabbit primary antibody at 4°C overnight, followed by incubation with a goat anti-rabbit secondary antibody conjugated to Alexa Fluor 488 (for NF-*κ*B p65) (1 : 1000, Abcam) or Alexa Fluor 594 (for ICAM-1) (1 : 1000, Abcam) for 2 h at room temperature. The slides were then mounted with one drop of mounting medium that contained DAPI. NF-*κ*B p65 and ICAM-1 were imaged using a confocal microscope. ICAM-1 was visualized as red and NF-*κ*B p65 was visualized as green, with nuclei as blue.

### 2.7. Measurement of Intracellular Reactive Oxygen Species

Intracellular ROS formation was determined with DCFH-DA (KeyGEN, Jiangsu, China). HUVECs were seeded in black 96-well plates. Subsequently, the cells were treated with TNF-*α* with or without indicated concentrations of hBD3 for 2 h. The cells were then washed with PBS and incubated with 20 *μ*M DCFH-DA for 30 min at 37°C in the dark. The fluorescence level was determined using a fluorescence microplate reader at an excitation wavelength of 485 nm and an emission wavelength of 530 nm.

### 2.8. F-Actin Staining

Human umbilical vein endothelial cells were cultured and treated with TNF-*α* as indicated. Then, the cells were fixed with 4% paraformaldehyde at room temperature for 20 min. After that, the actin cytoskeleton was stained with DyLight 488-phalloidin, and the nuclei were stained with DAPI. Stained HUVECs were visualized using a confocal microscope (Olympus FV 10i).

### 2.9. Statistical Analysis

Data are expressed as the means ± SD. Comparison between different treatments was performed using GraphPad PRISM software version 6.0 using one-way ANOVA with Tukey's post hoc test. Statistical significance was set at *p* values < 0.05.

## 3. Results

### 3.1. Effects of hBD3 on the Viability of TNF-*α*-Induced Endothelial Cells

**Figure 1:**
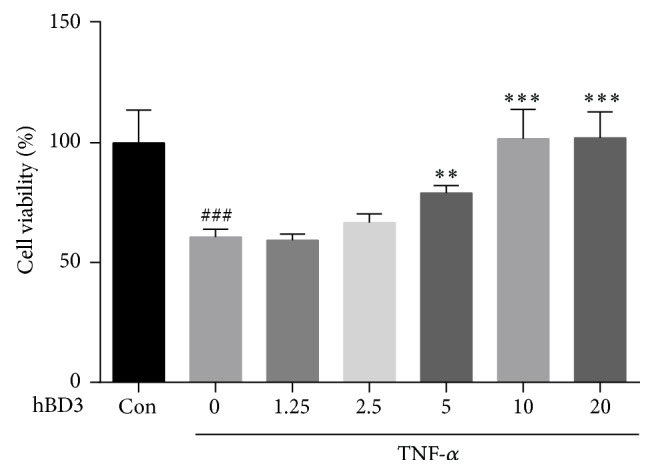
Effects of hBD3 on TNF-*α*-induced cell death. HUVECs were treated with TNF-*α* (40 ng/mL) and various concentrations of hBD3 for 24 h. The cell viability of HUVECs was determined with a CCK8 assay according to the instructions of manufacturers. Data are presented as the survival rate compared to the control group, which is defined as 100%. Values represent the means ± SD (*n* = 6). ^###^*p* < 0.001 compared with the control group. ^*∗∗*^*p* < 0.01, ^*∗∗∗*^*p* < 0.001 compared with TNF-*α* group.

Numerous studies have demonstrated that TNF-*α* treatment can impair endothelial cell viability and induce apoptosis. First, we evaluated the cytotoxic effects of TNF-*α* on HUVECs using a CCK8 assay. All data are expressed as the survival percentage of each group relative to the control group, which is defined as 100%. As shown in Figure 1, compared with the control group, TNF-*α* at a concentration of 40 ng/mL could markedly suppress the proliferation of HUVECs by nearly 50%. In addition, treatment with hBD3 at 5 *μ*g/mL and 10 *μ*g/mL markedly recovered TNF-*α*-impaired cell viability to about 79.1% and 101.7%, respectively. The results also suggested that hBD3 does not influence the viability of HUVECs.

### 3.2. Effects of hBD3 on the Production of Proinflammatory Mediators by Endothelial Cells Stimulated with TNF-*α*

**Figure 2:**
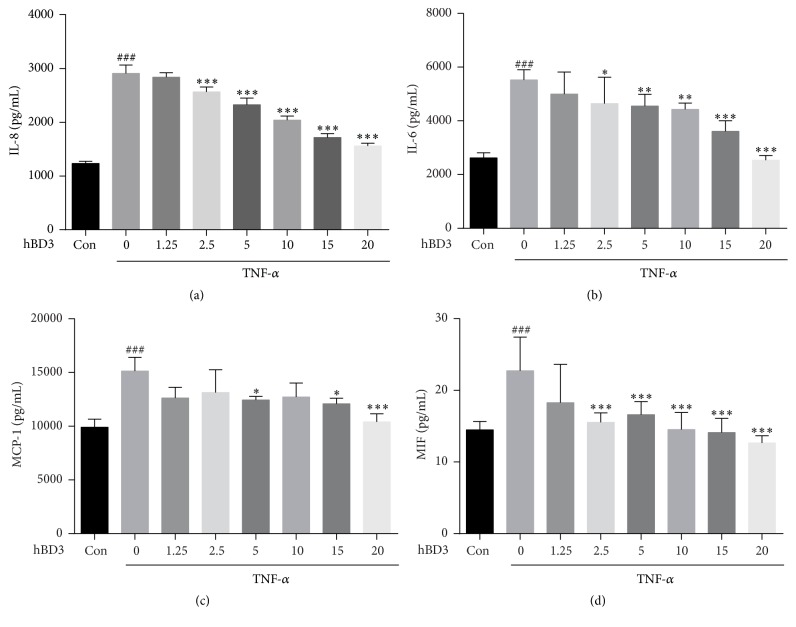
Effects of hBD3 on IL-8, IL-6, MCP-1, and MIF production in TNF-*α*-stimulated HUVECs. Cells were incubated with TNF-*α* (40 ng/mL) with or without various concentrations of hBD3 for 24 h. Then, the culture supernatants were collected by centrifugation and analyzed by ELISA according to the manufacturer's instructions. Data are expressed as the mean ± SD (*n* = 6). ^###^*p* < 0.001 compared with the control group. ^*∗*^*p* < 0.05, ^*∗∗*^*p* < 0.01, and ^*∗∗∗*^*p* < 0.001 compared with TNF-*α* group.

Next, we examined the effects of hBD3 on endothelial cell activation stimulated by TNF-*α*. Endothelial cells stimulated with TNF-*α* produce proinflammatory mediators, including interleukin- (IL-) 6, IL-8, and monocyte chemoattractant protein-1 (MCP-1), which cause more monocytes to be recruited. These proinflammatory mediators are reported to aggravate endothelial dysfunction. As shown in Figure 2(a), when the cells were treated with TNF-*α* for 24 h, IL-8 production in the supernatant was significantly elevated; however, with hBD3 intervention, IL-8 level was markedly and dose-dependently inhibited. The same trend of IL-6 and MCP-1 was also observed in Figures 2(b) and 2(c).

Macrophage migration inhibitory factor (MIF) is a key factor mediating the interactions between macrophages and endothelial cells [21]. It is considered to interact with other proinflammatory cytokines during inflammation. There is evidence showing it is a potent activator of macrophages released by activated endothelial cells. The release of MIF by HUVECs was markedly inhibited in response to hBD3 treatment (Figure 2(d)).

### 3.3. Effects of hBD3 on the Expression of Adhesion Molecules by HUVECs

**Figure 3:**
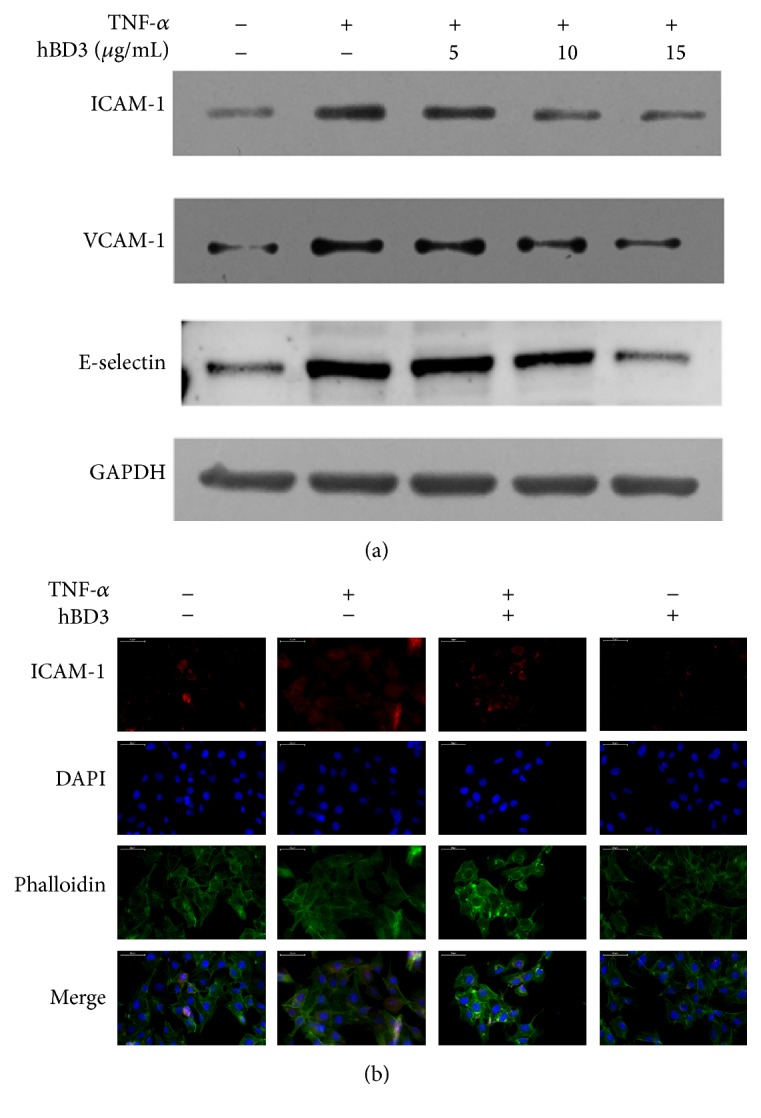
The effects of hBD3 on the expression of adhesion molecules in TNF-*α*-activated HUVECs. (a) HUVECs were incubated with TNF-*α* (40 ng/mL) with or without the indicated concentrations of hBD3 for 24 h (ICAM-1 and VCAM-1 analysis) or 4 h (E-selectin analysis). Whole cell lysates were extracted and analyzed by western blot using antibodies specific to VCAM-1, ICAM-1, and E-selectin. (b) Immunofluorescence analysis for ICAM-1 expression was conducted as mentioned above, and images were obtained using a fluorescence microscope (original magnification: 400x, scale bar 50 *μ*m).

Accumulated evidence supports a role for adhesion molecules in the progression of atherosclerosis. ICAM-1 and VCAM-1 are constitutively expressed on the surface of HUVECs, and E-selectin is only expressed on the activated endothelium [22]. The interaction of E-selectin and leukocytes mediates the rolling of leukocytes on the activated endothelium, which is the first event in firm adhesion. TNF-*α* is known to transiently upregulate the expression of adhesion molecules, such as ICAM-1 and VCAM-1, in HUVECs [23]. We then evaluated the effects of hBD3 on the expression of adhesion molecules induced by TNF-*α*. As demonstrated in Figure 3(a), treatment with TNF-*α* (40 ng/mL) significantly enhanced the expression level of ICAM-1 (24 h), VCAM-1 (24 h), and E-selectin (4 h). In addition, hBD3 treatment dose-dependently prevented the upregulation of ICAM-1, VCAM-1, and E-selectin levels by TNF-*α*. The immunofluorescence staining assay also validated the inhibitory effects of hBD3 (15 *μ*g/mL) on ICAM-1 expression levels triggered by TNF-*α* (Figure 3(b)).

### 3.4. Effects of hBD3 on Monocyte Adhesion to Endothelial Cells

**Figure 4:**
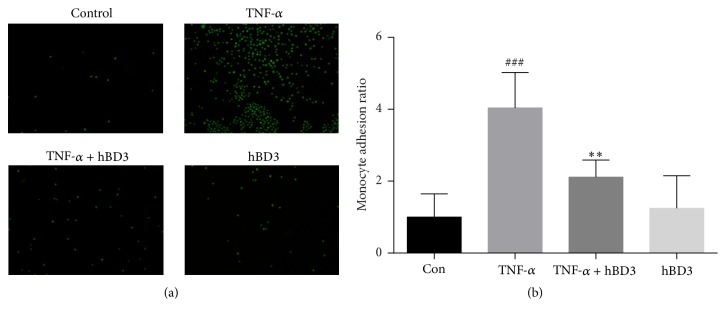
Effects of hBD3 on TNF-*α*-induced adhesion to THP-1 cells in HUVECs. HUVECs were stimulated with TNF-*α* (40 ng/mL) and the indicated concentrations of hBD3 for 24 h. After stimulation, HUVECs were cocultured with calcein-AM-labeled THP-1 cells for 30 min. (a) Images were captured using a fluorescence microscope (original magnification: 100x). (b) Adherent cells were measured using a microplate reader at excitation and emission wavelengths of 490 nm and 515 nm, respectively. The results were expressed as the mean ± SD, *n* = 3. ^###^*p* < 0.001 compared with the control group. ^*∗∗*^*p* < 0.01 compared with TNF-*α* group.

During the initiation of atherosclerosis, endothelial cells are activated and upregulate adhesion molecules and chemokine and cytokine secretion, which is beneficial for monocyte recruitment. Due to the significant decrease in cell surface adhesion molecules and proinflammatory mediators in hBD3-treated HUVECs, we investigated whether hBD3 could affect the attachment of immune cells to endothelial cells. To evaluate the effects of hBD3 on THP-1 monocyte adhesion to HUVECs in a TNF-*α*-stimulated proinflammatory environment, HUVECs were seeded and cultured to about 90% confluence and then stimulated with TNF-*α* (40 ng/mL) in the presence or absence of hBD3 (15 *μ*g/mL) for 24 h. Subsequently, HUVECs were cocultured with prelabeled THP-1 cells for 30 min. After removal of nonadherent THP-1 cells by gentle washing, images were captured under a fluorescence microscope. As shown in Figures 4(a) and 4(b), we observed that, compared with the control group, TNF-*α* treatment resulted in a 4-fold increase (*p* < 0.001) in THP-1 attachment to HUVECs. However, hBD3 intervention significantly decreased THP-1 adhesion to HUVECs (*p* < 0.01).

### 3.5. hBD3 Suppressed Activation of the NF-*κ*B and MAPK Pathways in HUVECs

NF-*κ*B is considered as an important regulator of the proinflammatory process and monocyte adhesion. To identify the probable molecular mechanism responsible for hBD3 involvement in the modulation of TNF-*α*-treated HUVECs, we first evaluated the classical NF-*κ*B signaling pathway. Here, we show that hBD3 strongly inhibits TNF-*α*-stimulated HUVECs via two distinct signaling pathways, an NF-*κ*B-dependent pathway and the MAPK pathway.

**Figure 5:**
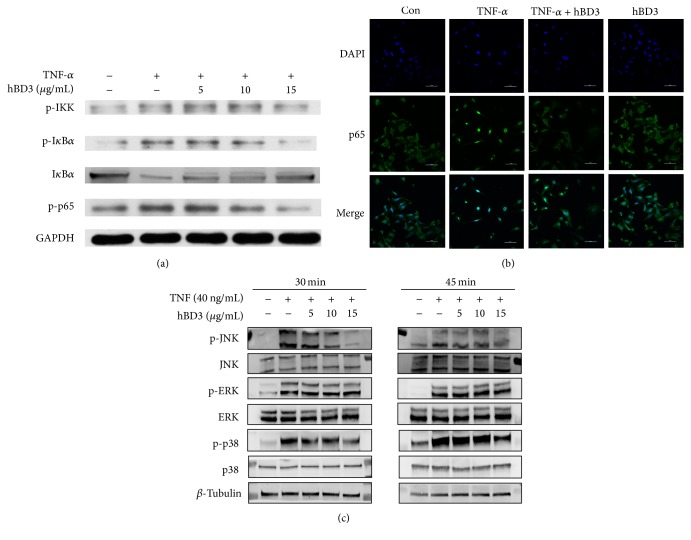
Effects of hBD3 on TNF-*α*-induced NF-*κ*B and MAPK activation in HUVECs. (a) HUVECs were incubated with TNF-*α* (40 ng/mL) with or without the indicated concentrations of hBD3 for 30 min. Whole cell lysates were centrifuged and analyzed with western blot using specific antibodies. (b) Immunofluorescence analysis for NF-*κ*B p65 localization was conducted as mentioned above, and images were captured using a confocal laser scanning microscope system (Nikon A1, Japan) (original magnification: 400x, scale bar 50 *μ*m). (c) HUVECs were incubated with TNF-*α* (40 ng/mL) with or without the indicated concentrations of hBD3 for 30 min and 45 min. Whole cell lysates were centrifuged and analyzed with western blot using specific antibodies.

We examined key factors in the NF-*κ*B pathway with western blot. Activation of NF-*κ*B required the phosphorylation and degradation of I*κ*B. As shown in Figure 5(a), degradation and phosphorylation of I*κ*B were significantly suppressed by the addition of hBD3. The effective role of hBD3 to prevent phosphorylation of p65 subunit was also observed. In addition, phosphorylated IKK*α*, which is the upstream modulator in the NF-*κ*B pathway, was also inhibited by hBD3. To validate this result, immunofluorescence staining was used to determine NF-*κ*B nuclear translocation. As shown in Figure 5(b), NF-*κ*B p65 was predominantly located within the cytoplasm in unstimulated cells. With TNF-*α* treatment, p65 translocated to the nucleus within 30 min. However, treatment with hBD3 at 15 *μ*g/mL led to retention of the p65 subunit in the cytoplasm of TNF-*α*-stimulated cells.

We then investigated the role of the MAPK pathway in this process. The key components of the MAPK pathway, p38, ERK, and JNK were evaluated with a western blot. As shown in Figure 5(c), treatment of TNF-*α* resulted in a significant increase of phosphorylation in the MAPK pathway. The phosphorylation of p38 and JNK was significantly and concentration-dependently suppressed by hBD3 at 30 min, while the phosphorylation of ERK was not altered. The cell lysates obtained at 45 min also validated this result.

### 3.6. Effects of hBD3 on the Activation of Caspases and Bcl-2 Expression in HUVECs

**Figure 6:**
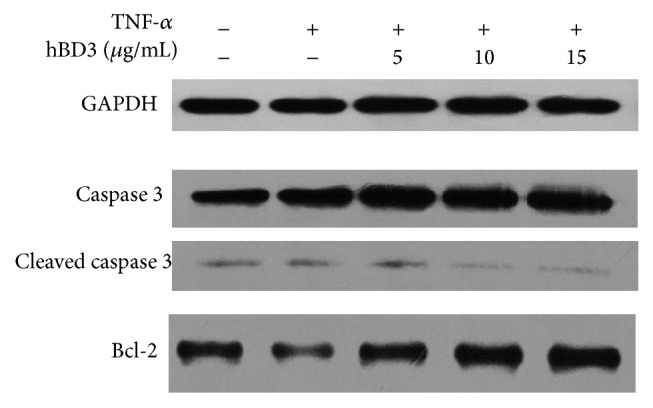
hBD3 inhibits HUVEC apoptosis stimulated by TNF-*α*. After incubation with TNF-*α* (40 ng/mL) and different concentrations of hBD3 for 6 h, the protein levels of cleaved caspase-3 and *β*-actin in HUVECs were detected with an immunoblot assay.

TNF-*α* has been reported to stimulate apoptosis in HUVECs. In addition, the cleavage of caspase-3 is a key step in TNF-*α*-induced cell death. Before activation, caspase-3 exists as an inactive form. After TNF-*α*-treatment, caspase-3 was cleaved to the active form. The activation of caspases is considered one mechanism of apoptosis. Therefore, we investigated the effects of hBD3 on the activation of caspases. HUVECs were treated with TNF-*α* (40 ng/mL) for 6 h. The expression levels of caspase-3 were examined with western blot. Western blot analysis revealed that the expression levels of cleaved caspase-3 significantly increased following TNF-*α* treatment, while hBD3 markedly suppressed this phenomenon. Meanwhile, we examined the expression level of Bcl-2. As shown in Figure 6, there was a clear decrease in the expression of Bcl-2 after treatment with TNF-*α*. These results suggested that hBD3 inhibited the apoptosis of HUVECs stimulated by TNF-*α*.

### 3.7. hBD3 Suppressed TNF-*α*-Induced ROS Formation in HUVECs

**Figure 7:**
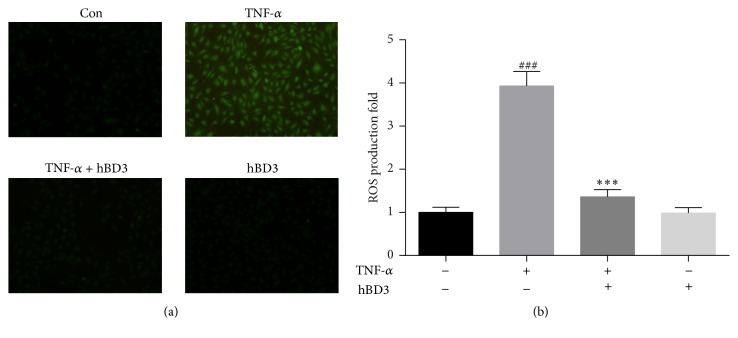
Inhibitory effects of hBD3 on TNF-*α*-induced ROS formation in HUVECs. HUVECs were incubated with TNF-*α* (40 ng/mL) and hBD3 (15 *μ*g/mL) for 2 h. Intracellular ROS accumulation was determined by observation under a fluorescence microscope (a) or fluorescence measurement with a microplate reader (b) after incubation with the ROS detector DCFH-DA for 30 min (original magnification: 400x). Values represent the mean ± SD. ^###^*p* < 0.001 compared with the control group; ^*∗∗∗*^*p* < 0.001 compared with TNF-*α* alone.

It has been suggested that the enhanced production of intracellular ROS can lead to cell apoptosis. To measure the potential modulation of apoptosis and oxidative stress by hBD3, the production of ROS was evaluated for fluorescence intensity in HUVECs. As revealed in Figure 7(a), HUVECs stimulated with TNF-*α* produced an intracellular burst of ROS (about 3.9-fold change). The intracellular ROS production in HUVECs was significantly suppressed when treated with hBD3. However, the basal level of ROS was not altered by treatment with hBD3 alone.

### 3.8. hBD3 Repressed TNF-*α*-Induced F-Actin Reorganization in HUVECs

**Figure 8:**
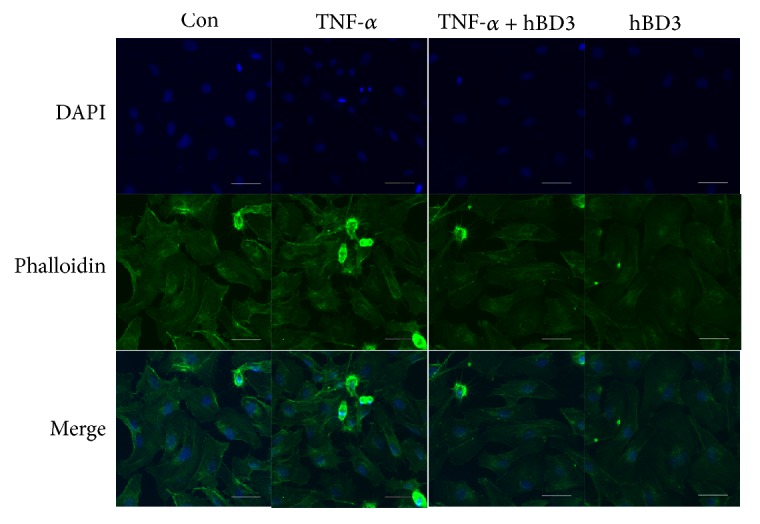
Effects of hBD3 on TNF-*α*-induced F-actin rearrangement. HUVECs were incubated with TNF-*α* (40 ng/mL) with or without hBD3 (15 *μ*g/mL) for 24 h. After that, cells were washed with PBS. F-actin was stained with DyLight 488-phalloidin for 15 min in the dark according to the manufacturer's instructions. Images were obtained using a confocal microscope (Olympus FV10i) (original magnification: 600x, scale bar 50 *μ*m).

TNF-*α* is reported to have the ability to alter the permeability and morphology of endothelial cells. To investigate the role of hBD3 in TNF-*α*-induced endothelial function, HUVECs were treated with TNF-*α* (40 ng/mL) in the presence or absence of hBD3 (15 *μ*g/mL) for 24 h. Then, the cells were fixed and stained with phalloidin. As shown in Figure 8, stimulating HUVECs with TNF-*α* resulted in a significant increase in the formation of actin stress fibers. In addition, hBD3 treatment markedly reduced this alteration in HUVECs.

## 4. Discussion

Atherosclerosis is defined as a chronic inflammatory-fibroproliferative response of the vascular wall to various forms of inflammatory stimuli. Disruption of the endothelial cell barrier induced by proinflammatory cytokines, such as TNF-*α*, is the initiating step of this disease.

Endothelial cells maintain vascular homeostasis via the secretion of several vasoactive factors, and impairment of barrier integrity leads to the development of vascular inflammatory diseases, such as atherosclerosis. As a monolayer in direct contact with the bloodstream, the vascular endothelium is the principal physiological target of the proinflammatory actions of TNF-*α* and IL-1. Extensive evidence has suggested that the endothelium is not simply the inner cellular lining of blood vessels [24]. Beyond being passively targeted, endothelial cells are believed to be profoundly involved in this inflammatory process, especially in the regulation of effector cells, such as monocytes and lymphocytes. Therefore, the endothelium may act as a therapeutic target due to its critical role in the inflammatory process [25].

Macrophage migration inhibitory factor (MIF) is one of the most important cytokines produced by endothelial cells to control the accumulation of effector cells [26]. It is recognized as an effective activator of macrophages, participating in the process of recruitment and the accumulation of macrophages at local inflammatory sites. As monocyte adhesion to the endothelium is a crucial step in the early stages of atherosclerosis, MIF is considered to be involved in the pathogenesis of atherosclerosis and is closely related to plaque stability [27]. Elevated MIF expression was observed in response to some immunostimulants in HUVECs [28]. In our experiment, the production of MIF was markedly increased under TNF-*α* stimulation in HUVECs, while treatment with hBD3 dose-dependently reduced MIF expression. This result was also confirmed by the effect of hBD3 in inhibiting the adhesion of monocytes to endothelial cells.

A prominent feature of TNF-*α*-induced endothelial cell injury is the excess generation of intracellular ROS. Moderate levels of ROS have been reported to participate in the physiological functions of cells, while the overproduction of intracellular ROS is closely associated with the progression of atherosclerosis [29, 30]. Oxidative stress activates endothelial cells, causing cell injury, apoptosis, and enhanced monocyte adhesion. In the present study, treatment with hBD3 attenuated ROS production in HUVECs stimulated with TNF-*α*.

The binding of TNF-*α* to cell surface receptors triggers multiple signaling events, including NF-*κ*B and MAPK pathway. NF-*κ*B has been demonstrated to be a key transcription factor in this signaling pathway. Activation of NF-*κ*B is closely associated with the initiation and progression of atherosclerosis. In resting cells, NF-*κ*B is maintained in an inactive form within the cytoplasm by the inhibitor of *κ*B (I*κ*B) family but enters the nucleus in response to external stimuli, such as TNF-*α*. I*κ*B kinase- (IKK-) dependent phosphorylation and the subsequent ubiquitination and degradation of I*κ*B make NF-*κ*B free to translocate to the nucleus and induce downstream transcription [31]. NF-*κ*B has been identified as an important target in the treatment of atherosclerosis. Our data revealed that hBD3 markedly reduced the phosphorylation of I*κ*B. Additionally, the p65 subunit of NF-*κ*B is constitutively phosphorylated at a low level in unstimulated conditions, and treatment with inflammatory triggers, LPS and TNF-*α*, for example, results in further phosphorylation. The total level of phosphorylation of p65 was also suppressed by the addition of hBD3.

We also investigated the phosphorylation of IKK-*α*/*β*, which is an upstream signaling molecule of NF-*κ*B. The results demonstrated that hBD3 inhibited IKK-*α*/*β* phosphorylation 30 min after TNF-*α* stimulation. In addition, the inhibition of ROS generation also contributes to the decrease in NF-*κ*B activation, as NF-*κ*B activation by ROS through the IKK pathway has been suggested [32]. The downregulation of cell adhesion molecules in HUVECs by hBD3 might be mediated through inhibition of the NF-*κ*B pathway, as the TNF-*α*-mediated increase in ICAM-1 and VCAM-1 expression is mediated through the NF-*κ*B pathway, not the MAPK pathway [33].

Ample evidence has documented intensive crosstalk between the NF-*κ*B and MAPK signaling pathways. The MAPK pathway is required for NF-*κ*B activation in TNF-*α*-mediated inflammation [34]. In addition, the JNK pathway was suggested to promote cell apoptosis [35]. hBD3 was reported to rapidly enter the cells and impact TLR signaling pathway associated with MyD88 and TRIF [36]. In this case, there is also possibility that hBD3 inhibits TNF signaling via interaction with the upstream molecules in the signaling pathway. Further research needs to be done to elucidate this problem.

Human *β*-defensin 3 is a multifunctional effector molecule involved in a startling range of cellular processes. First, as an antimicrobial peptide, hBD3 exhibits broad antimicrobial activity against Gram-positive and Gram-negative bacteria, fungi, and viruses [37]. Second, it was demonstrated to have immunomodulatory effects due to its biphasic effect in the host immune response. It can be either proinflammatory or anti-inflammatory, depending on the circumstances [38, 39]. In addition, the beneficial effects of hBD3 on wound healing were also reported [40]. We previously demonstrated that hBD3 significantly inhibited the progression of early-stage atherosclerotic lesions, and this effect was correlated with downregulation of macrophage inflammation. To date, there is no study focusing on the effects of hBD3 on TNF-*α*-induced endothelial inflammation. In the present study, our data extend the protective effects of hBD3 to TNF-*α*-induced endothelial cell injury.

Owing to their strong antimicrobial activity, low molecular weight, and immunogenicity, several researchers and studies are focusing on the clinical use of antimicrobial peptides as a new class of antibiotics [41], and some of them are already in the clinical development stage [42]. However, *β*-defensins had limited success in clinical application. The reasons include the high synthesis cost and short half-lives in blood. Cationic peptides are not stable in the serum due to high susceptibility to enzymatic degradation. hBD3 was observed to be completely digested in contact with simulated gastric fluid [43]. One solution to the limitations is gene delivery of hBD3. Another way is to develop a structurally modified peptide of hBD3 with certain biological activity. Notable examples include a C-terminus peptide and a linear hBD3 peptide with anti-inflammatory and antifungal activities [44–46]. This may provide new therapeutic approaches to harness the pharmaceutical potential of hBD3.

However, the endothelial cells we used in this study were obtained from the human umbilical vein, not aortas, which are the main location forming atherosclerotic plaques. The difference in physiology and shear stress between veins and arteries might contribute to heterogeneity between ECs in veins and aortas [47–49]. In addition, some differences in the immune response to proinflammatory cytokines, such as TNF-*α*, might exist. Further confirmation in animal models should be performed to uncover the underlying molecular mechanisms. In addition, cell density was identified to have an impact on the immune response of HUVECs [50].

## 5. Conclusion

In summary, the results of this study demonstrate that TNF-*α*-induced endothelial cell activation can be reversed by treatment with hBD3. Furthermore, the anti-inflammatory effects of hBD3 in TNF-*α*-treated HUVECs seem to be closely related to downregulation of NF-*κ*B pathway and MAPK pathway.
